# A Microbiota-Dependent Response to Anticancer Treatment in an *In Vitro* Human Microbiota Model: A Pilot Study With Hydroxycarbamide and Daunorubicin

**DOI:** 10.3389/fcimb.2022.886447

**Published:** 2022-06-01

**Authors:** Claire Amaris Hobson, Lucile Vigué, Mélanie Magnan, Benoit Chassaing, Sabrine Naimi, Benoit Gachet, Pauline Claraz, Thomas Storme, Stephane Bonacorsi, Olivier Tenaillon, André Birgy

**Affiliations:** ^1^ IAME, UMR 1137, INSERM, Université de Paris, AP-HP, Paris, France; ^2^ INSERM U1016, team “Mucosal microbiota in chronic inflammatory diseases”, CNRS UMR 8104, Université de Paris, Paris, France; ^3^ Service de pharmacie-Hôpital Robert Debré, DMU PRISME, AP-HP. Nord, Paris, France; ^4^ Laboratoire de Microbiologie, Hôpital Robert Debré, AP-HP, Paris, France

**Keywords:** gut microbiota, *in vitro* microbiota model, MBRA, hydroxycarbamide, daunorubicin, anticancer treatment and bacteria

## Abstract

**Background:**

Anticancer drug efficacy is linked to the gut microbiota’s composition, and there is a dire need to better understand these interactions for personalized medicine. *In vitro* microbiota models are promising tools for studies requiring controlled and repeatable conditions. We evaluated the impact of two anticancer drugs on human feces in the MiniBioReactor Array (MBRA) *in vitro* microbiota system.

**Methods:**

The MBRA is a single-stage continuous-flow culture model, hosted in an anaerobic chamber. We evaluated the effect of a 5-day treatment with hydroxycarbamide or daunorubicine on the fecal bacterial communities of two healthy donors. 16S microbiome profiling allowed analysis of microbial richness, diversity, and taxonomic changes.

**Results:**

In this host-free setting, anticancer drugs diversely affect gut microbiota composition. Daunorubicin was associated with significant changes in alpha- and beta-diversity as well as in the ratio of Firmicutes/Bacteroidetes in a donor-dependent manner. The impact of hydroxycarbamide on microbiota composition was not significant.

**Conclusion:**

We demonstrated, for the first time, the impact of anticancer drugs on human microbiota composition, in a donor- and molecule-dependent manner in an *in vitro* human microbiota model. We confirm the importance of personalized studies to better predict drug-associated-dysbiosis *in vivo*, linked to the host’s response to treatment.

## 1 Introduction

Cancer incidence increases yearly, with 18 million new diagnostics worldwide in 2018 and over 19 million in 2020 (Sung et al., 2021). Accordingly, the prescription of anticancer drugs, associated with highly heterogeneous efficacy and toxicity among cancer patients, is also increasing around the globe.

The frequent digestive symptoms reported after these treatments (diarrhea, mucositis) support a significant alteration of the human gut microbiota, leading to drug-associated-dysbiosis; however, no studies monitored the drug concentration in the feces. Yet, though neglected for decades, this induced modification in the gut microbiota composition may have major consequences, as besides its implication in human pathology (Nicholson et al., 2012; Sivaprakasam et al., 2016; Ratajczak et al., 2019; Bilotta et al., 2021).

Anticancer drugs have been developed to target cancer cells; however, they are nonspecific, and some even have well-known antimicrobial properties (Hamilton-Miller, 1984; Bodet et al., 1985; Guðmundsdóttir et al., 2021). For the majority, these drugs are antimetabolites (Gieringer et al., 1986), induce DNA damage (Babudri et al., 1984), or interfere with the replication cycle (Davies et al., 2009; Wozniak and Simmons, 2021). Importantly, if bacteria are targeted, their ecosystem, such as the gut, is also threatened (Rashidi et al., 2019). Indeed, anticancer treatments alter the gut microbial diversity and richness (van Vliet et al., 2009; Montassier et al., 2014).

Evidence is growing on the importance of the gut microbiota composition in the host’s response to anticancer treatment (Iida et al., 2013; Viaud et al., 2013; Chaput et al., 2017; Routy et al., 2018). The gut microbiota could even be used to monitor cancer progression (Pope et al., 2017) as a predicator of the response to anticancer therapies (Chaput et al., 2017; Routy et al., 2018) or infectious anticancer-treatment-related side effects (Dubin et al., 2016; Montassier et al., 2016; Aarnoutse et al., 2019). Modulation of the gut microbiota to influence the host’s response to anticancer drugs is, therefore, one of the main therapeutic issues (Jia et al., 2008; Viaud et al., 2013; Sivan et al., 2015; Alexander et al., 2017; Ichim et al., 2018; Aarnoutse et al., 2019; Severyn et al., 2019), and personalized medicine appears as an opportunity to optimize a patient’s treatment while limiting the side effects (Montassier et al., 2016; Alexander et al., 2017).

All these interactions and their potential dramatic effects on a patient’s health are calling for a deeper characterization and, particularly, a clarification of the impact of chemotherapy on gut microbiota composition. Indeed, most of the studies evaluating the impact of anticancer drugs are designed *in vivo* in patients receiving concomitant treatments such as anticancer drug combinations or the frequent treatment with antimicrobials.

While the intestinal microbiota appears to play a role in drug efficacy and therapeutic response, the exact mechanism remains unknown. In clinical and preclinical approaches, it is difficult to separate direct responses due to microbiota–drug interactions from tripartite ones, including host responses to host–drug–microbiota. In that respect, using an *in vitro* microbiota model allows the exclusion of the confounding factors associated with the gut microbiota disturbances inherent to the host or its environment (Chassaing et al., 2017; Jin et al., 2017) and allows to narrow down the focus on only gut microbiota and anticancer drugs treatments. Furthermore, these models offer the advantages of low ethical constraints, an unlimited number of replicates, and high reproducibility, and thus allow testing many different conditions.

Along those lines, we evaluated the relevance of the MiniBioReactors Array (MBRA) *in vitro* human microbiota model, which allows a dynamic, stable culture of human-derived microbiota under anaerobic conditions (Auchtung et al., 2015; Naimi et al., 2021) to study specifically anticancer drug–microbiota interactions.

Facing many anticancer drugs to test, we decided to evaluate the impact of two anticancer drugs used in leukemia and other hematologic malignancies, hydroxycarbamide and daunorubicin. To assess the direct effect of these two drugs on the human gut microbiota composition, we simulated an anticancer treatment using the MBRA model, which was inoculated with fecal samples of two health donors followed over 5 days of treatment.

## 2 Materials and Methods

### 2.1 Experimental Design

**Figure 1:**
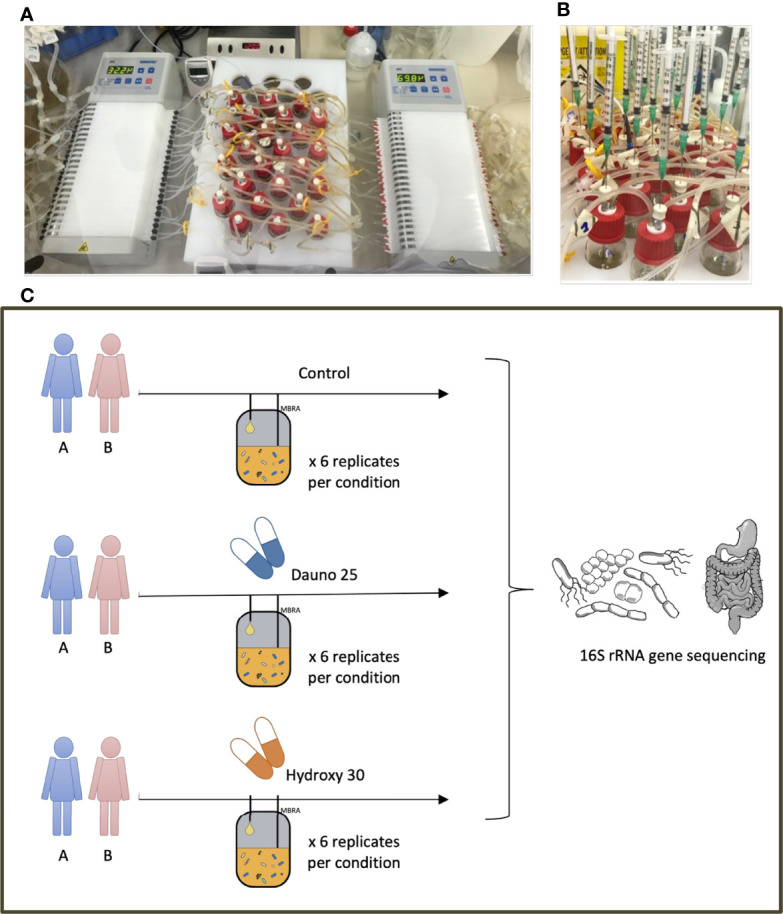
MBRA model and experimental design. **(A)** The MBRA model, the input and output pumps, and the 24 culture chambers placed on a magnetic stirring plate. **(B)** The treatment administration, simultaneously, directly in the culture chambers. **(C)** The experimental design and the 6 conditions evaluated in this work.

We conducted an *in vitro* experiment using the MBRA system, hosted in an anaerobic chamber at 37°C, as described previously by Auchtung et al. (2015) and Naimi et al. (2021). The MBRA system consists of 24 independent chambers, enabling a 15-ml culture volume to be maintained throughout the experiment by two peristaltic pumps. The whole system is stationed on a magnetic stirring plate to mimic intestinal peristalsis. All the components of the system are stored in the anaerobic chambers at least 48 h prior to the experiment, including the culture media, the BioReactor Medium (BRM) (Auchtung et al., 2015), placed 72 h before the experiment in the chamber (**Figure 1**).

### 2.2 Preparation of Fecal Samples

Human fecal samples were collected from two healthy donors belonging to the same age class (30 +/− 5 years old). None had consumed antibiotics nor had any medical issues within the previous 6 months. In agreement with the INSERM ethics regulation and with the declaration of Helsinki, all received clear information and consented. Fecal samples were directly collected in a sterile container and placed in anaerobic conditions. Within 24 h after emission, the samples were subsequently subdivided into sterile vials (under anaerobic conditions) and stored at −80°C until use.

### 2.3 Inoculation and Sample Collection

Fecal samples were processed as previously described (Auchtung et al., 2015; Naimi et al., 2021). After a 16–18-h resting period, the flow rate was at 1.875 ml/h, corresponding to an 8-h retention time. The experiment was divided into three experimental phases: a pretreatment phase for microbiota stabilization {from resting period [initiation (I)] to stabilization (S)}, a treatment phase (1 daily dose, for 5 consecutive days, samples “T”), and a posttreatment phase (starting 24 h after the last dose (T5) until the end of the experiment, 96 h after the last treatment dose, “PT”) (Naimi et al., 2021).

### 2.4 Anticancer Treatments

To study the effect of anticancer molecules on gut microbiota composition, we selected among the most used molecules an anthracycline aminoglycoside [daunorubicin 25 mg/L (dauno25)] (Carol et al., 2015) and an alkylating agent [hydroxycarbamide 30 mg/L (hydroxy30)] (Estepp et al., 2018) **Figure 1**. Tested concentrations were based on the literature, which corresponded to plasmatic or fecal concentrations. Treatment was administered directly in each chamber, daily, for 5 consecutive days. Six replicates were performed per condition on 2 different fecal samples from healthy donors. The evolution of the 48 microcosms (treatment versus control * 2 drugs * 2 donors * 6 replicates per condition) was followed through the characterization of the 16S rRNA gene diversity over 8 time points resulting in the analysis of 384 samples.

### 2.5 Sample Processing and Sequencing

#### 2.5.1 DNA Extraction

We used the DNeasy Power Soil Pro 250 kit^®^ (Qiagen) for total DNA extraction, following the manufacturer’s protocol. We collected 100 µl of frozen MBRA-fecal samples from 8 time points (I-S-T1-T2-T3-T4-T5-PT) for gut microbiota composition analysis. We subsequently used the Qiacube^®^ High-Throughput 96-sample robot for DNA extraction.

### 2.6 16S rRNA Gene Sequencing

Intestinal gut microbiota composition in collected samples was analyzed using 16S rRNA gene sequencing (Yarza et al., 2014; Rashidi et al., 2019), targeting the V4 hypervariable region (Bukin et al., 2019). The manufacturer’s instructions for the library preparation were followed, using KAPA HiFi HotStart ReadyMix (Roche Laboratories, Basel, Switzerland). Briefly, the amplification of the 16S region (“PCR 1”) was performed with the 515 forward primer (5′-GTGCCAGCMGCCGCGGTAA-3′) and the 806 reverse primer (5′-GGACTACHVGGGTWTCTAAT-3′) (Caporaso et al., 2011). The multiplexing step of the samples on the 16S amplified regions (“PCR 2”) required the Nextera^®^ Index Kit and Nextera^®^ XT Index Kit V2 Set D. PCRs products were visualized by gel electrophoresis and quantified using the Qubit HS^®^ normalized to 4 nM for pooling and sequencing. 16S rRNA gene sequencing was performed with the Illumina MiSeq technology (paired-end reads 2 * 250 bp).

### 2.7 Data Analysis

16S rRNA gene sequences were analyzed using QIIME2-2020.8 software for microbiome bioinformatics analysis (Bolyen et al., 2019). The samples were denoised with the DADA2 (*via* “q2-dada2”) pipeline (Callahan et al., 2016). The reads were subsequently aligned with mafft (Katoh et al., 2002) (*via* “q2-alignment”) and phylogeny was constructed with fasttree-2 (Price et al., 2010) (*via* “q2-phylogeny”). Alpha diversity (diversity within each chamber) was analyzed with the Shannon Index, while beta diversity (diversity between each chamber) was represented by Jaccard analysis (using “q2-diversity”).

More specifically, for alpha diversity, we estimated a relative deviation for each chamber from the before-treatment baseline. Subsequently, a Permutational multivariate analysis of variance (PERMANOVA) (function aovp from the “lmPerm R” package) was used in which *p*-values are based on permutations. Up to 5,000 permutations were made (less when the *p*-value converged to a high value), *p*-values around 0.05 and below are reported, and minimal value is 1e−4. Because alpha diversity globally declines with time, we used time as a cofactor (treated as a factor) in the PERMANOVA. To better consider the decrease in diversity with time, we also performed a linear model with *p*-values estimated by permutation (function lmp from the “lmPerm R” package, Alpha_diversity~time+treatment). We used the same approach to study beta diversity.

Diversity analysis required the samples to be prior rarefied to 10,000 and 7,500 sequences per sample in the hydroxycarbamide and daunorubicin experiments, respectively. We assigned taxonomy to all amplicon sequence variants using a Naive Bayes classifier trained on the Greengenes reference database 13_8 (using “q2-feature-classifier”) (McDonald et al., 2012). Rarefied data were used for counts. Last, we estimated the Firmicutes to Bacteroidetes ratio (F/B), including the times during and after treatment. All generated data visualizations were performed using Python3. R software was used to perform the statistical tests.

## 3 Results

### 3.1 Donor and Time Impact Microbiota Composition in the MBRA System

Using the 16S microbiome profiling and the MBRA *in vitro* human gut system, we quantified the *in vitro* impact of hydroxycarbamide and daunorubicin on gut microbiota composition. During the stabilization phase (S), differences between the treated and nontreated chambers (through Firmicutes to Bacteroidetes ratio and alpha diversity) were nonsignificant (*p* > 0.1 for donors A or B on either drug), and all 12 chambers per donor could be considered comparable.

**Figure 2:**
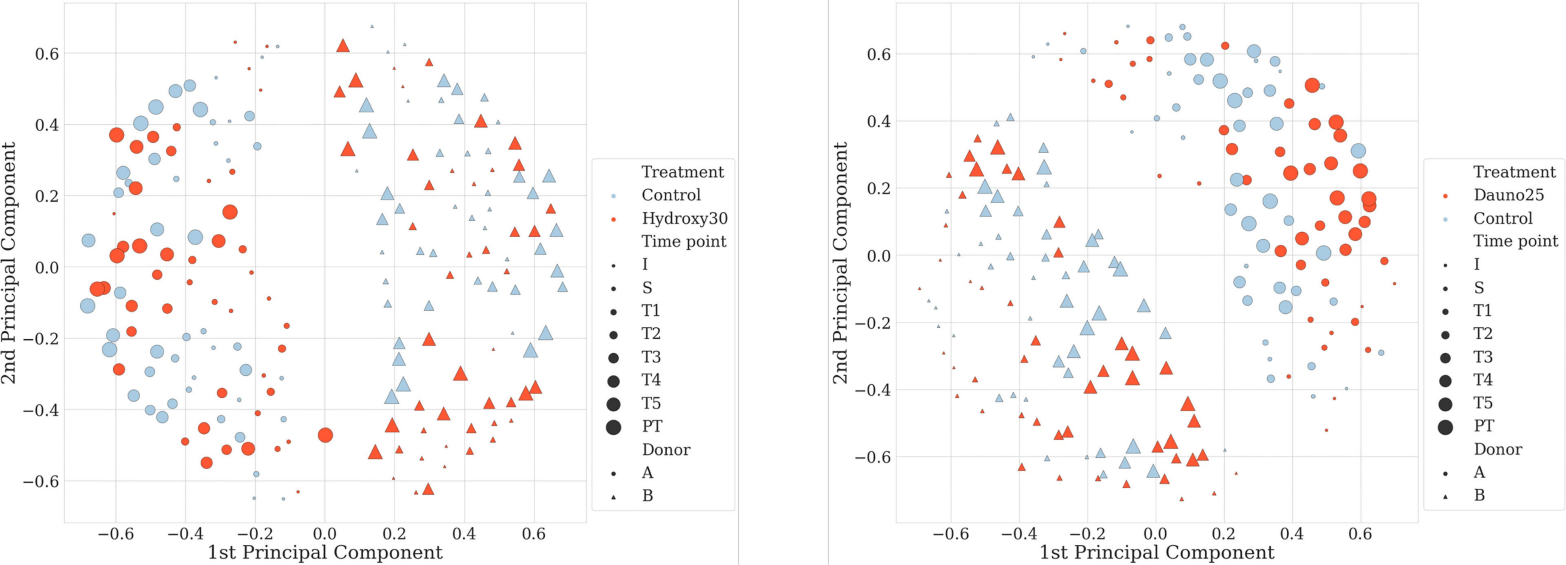
Principal coordinate analysis (PCoA) of the Jaccard metrics, with all donors, all conditions, and all time points. Dots represent donor A and triangles donor (B) Size dots/triangles are proportional to the time of the experiment; the bigger the later in the experiment. Figure on the left-hand side represents hydroxycarbamide and daunorubicin on the right-hand side.

Principal coordinate analysis (PCoA) of the Jaccard matrix from all time points, all conditions, and all donors showed that the two donors could be well distinguished throughout the experiment (**Figure 2**). No specific pattern associated with the treatment or the time could be evidenced in this analysis.

### 3.2 Impact of Treatment on Alpha and Beta Diversity

First, to study alpha diversity, a PERMANOVA was used in which *p*-values are based on permutations (minimal value 1e−4).

**Figure 3:**
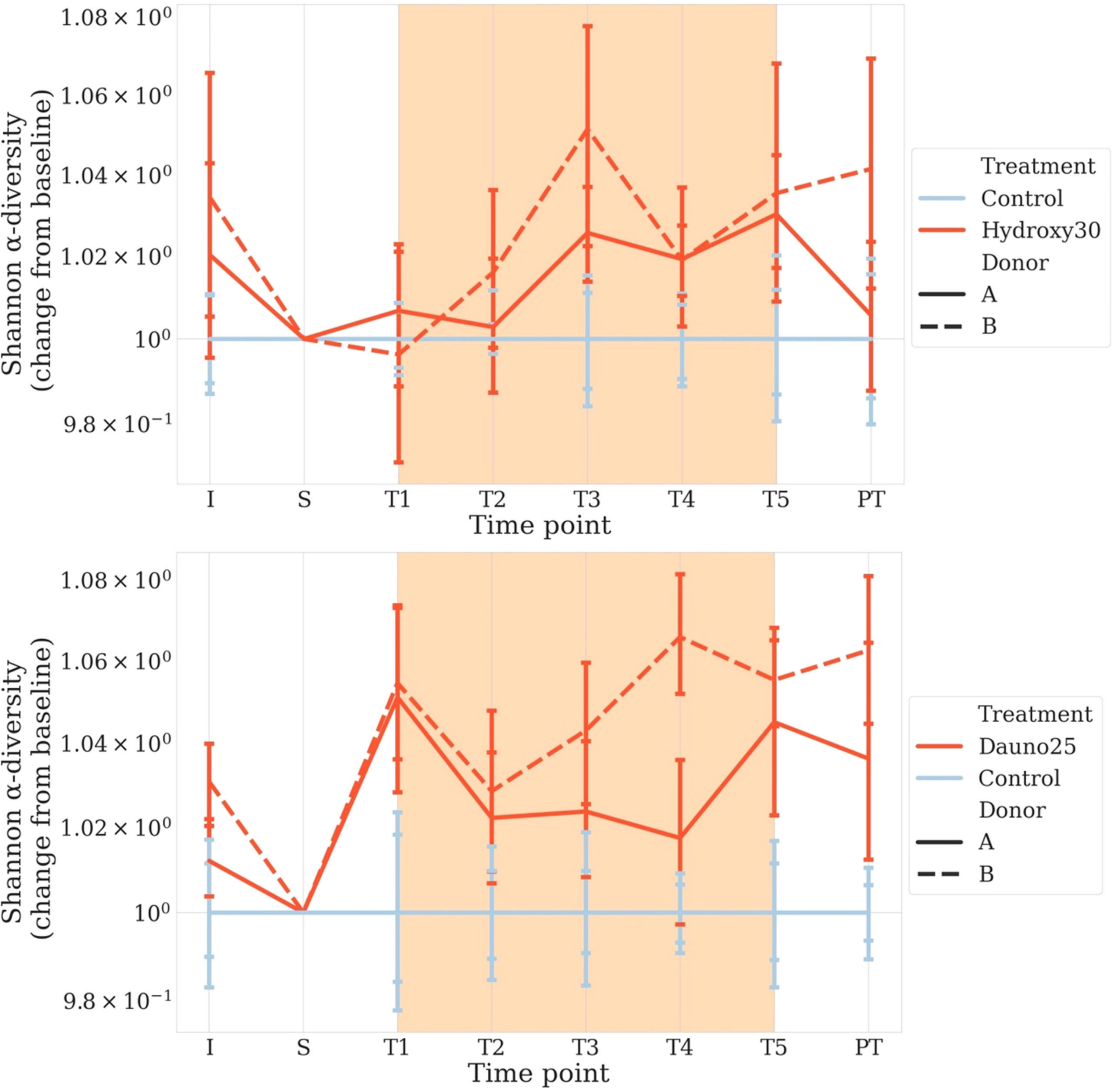
Change from baseline of the Shannon Diversity Index in donors A and B, in all conditions. S is considered the baseline. Solid lines represent donor (A), and dotted lines represent donor (B) Light blue represents the controls, and red represents the treated groups. Orange square represents the treatment period (average and 68% CI). All time points are normalized so that control chambers average at 1. The top figure represents hydroxycarbamide, and the bottom figure represents daunorubicin.

The daunorubicin-related disturbance in alpha diversity (Shannon Index) was significant whether we pooled data from both donors, singled out donor B, or to a lesser extent when we singled out data from donor A (*p* < 1e−4, *p* < 1e−4, and *p* = 0.0002, respectively) (**Figure 3**).

The hydroxycarbamide-related disturbance in alpha diversity was much less clear, with an overall effect of treatment (*p* = 0.004) and an effect for donor B (*p* = 0.02) but no effect for donor A (*p* = 0.07) (**Figure 3**).

Our results were confirmed by a linear model with *p*-values estimated by permutation considering the decrease in diversity through time. Results were consistent with the first approach with an effect of daunorubicine (A and B: *p* < 1e−4, B: *p* < 1e−4, A: *p* = 0.01) and a modest or lack of effect for hydroxycarbamide (A and B: *p* = 0.001, B: *p* = 0.02, A: *p* = 0.6).

For beta-diversity analyses, the same approach was used. We measured the Jaccard Index between the chambers before treatment and the later time points. For both drugs, results were consistent with the one observed with alpha diversity, but this time, the treatments induced a faster differentiation compared with controls.

**Figure 4:**
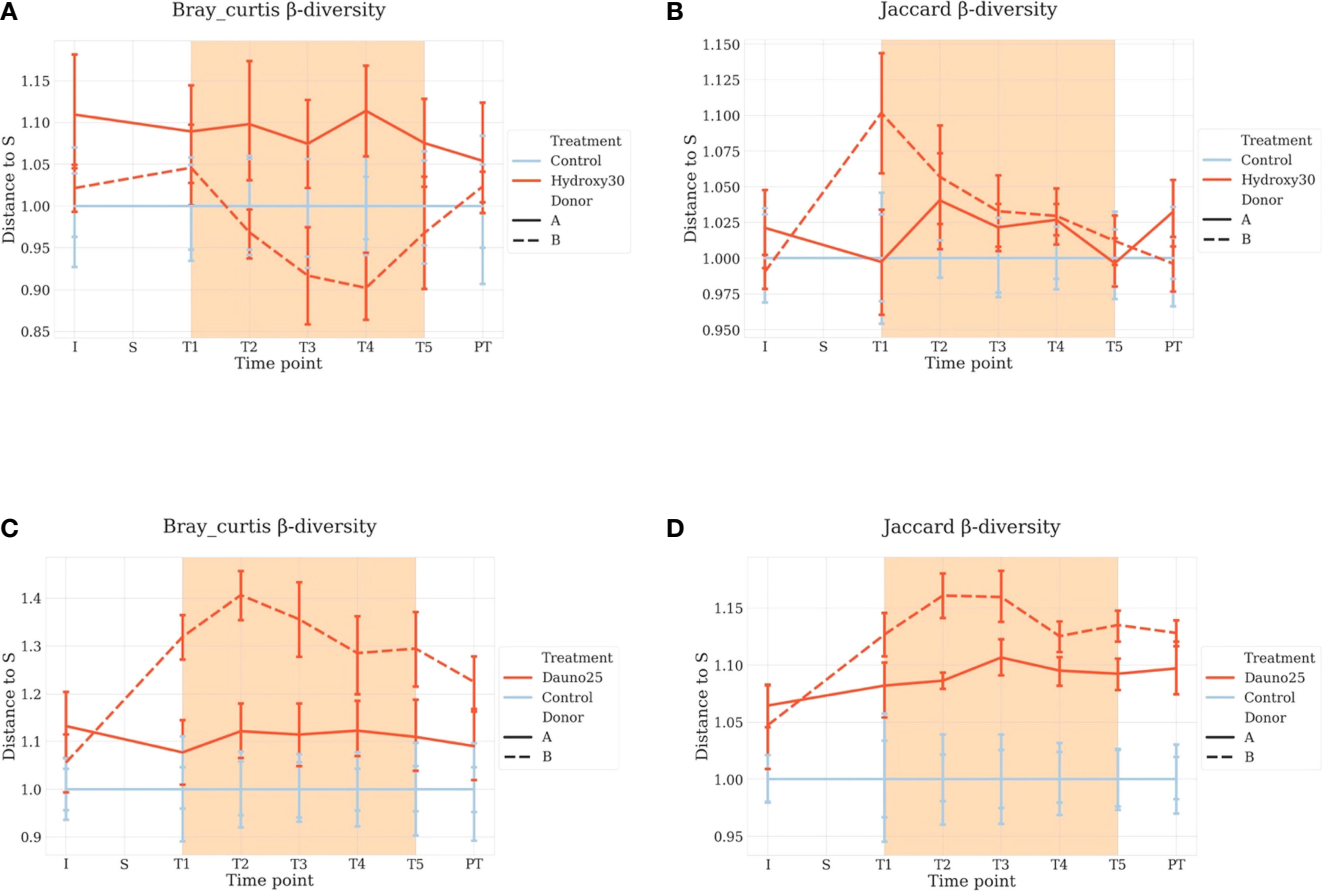
Beta diversity Bray–Curtis **(A–C)** and Jaccard metrics **(B–D)** representation. Each condition is compared with the control at the same time point, and S is baseline. Light blue represents the controls, and red represents the treated groups. Orange square represents the treatment period (average and 68% CI). All time points are normalized so that control chambers average at 1. **(A, B)** Hydroxycarbamide; **(C, D)** daunorubicin.

Daunorubicin was associated with a significant effect in all cases (aovp: *p* < 1e−4, lmp: *p* < 1e−4 for A, B, and A and B). Hydroxycarbamide was associated with a mild effect in donor B (aovp: *p* = 0.03, lpmp: *p* = 0.02) and the combination of donors (aovp: *p* = 0.014, lmp: *p* = 0.015), but not in donor A (aovp: *p* = 0.2, lmp: *p* = 0.4) (**Figure 4**).

Using Bray–Curtis metrics, similar results were found. The overall effect of treatment was not found to be significant for hydroxycarbamide (*p* = 0.23) but was marginally significant for donor A (*p* = 0.04).

For diversity representations (**Figures 3**, **4**), we normalized all chambers so that the control average to 1 to highlight variation in the treated chambers. In this way, we normalized the interindividual variability observed between donors for the untreated chambers. We calculated the distance to S (stabilization period before treatment) for each condition, meaning each chamber is its own control, and all time points are compared with S, allowing us to study variations within each culture chamber (**Figure 4**).

### 3.3 Impact of Treatment on Bacterial Communities

**Figure 5:**
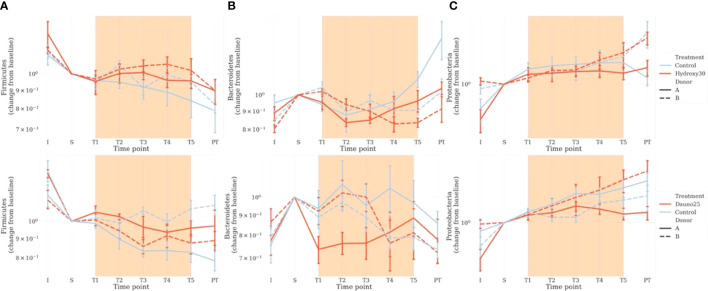
Impact of the treatments on the main 3 phyla of interest: Firmicutes **(A)**, Bacteroidetes **(B)**, and Proteobacteria **(C)**. Counts were estimated from rarefied tables. Fecal bacterial composition was analyzed after 16S rRNA gene sequencing. Solid lines represent donor A, and dotted lines represent donor **(B)** Light blue represents the controls, and red represents the treated groups. Orange square represents the treatment period (average and 68% CI). The top figure represents hydroxycarbamide, and the bottom figure represents daunorubicin.

Taxonomic analysis at the phylum level enabled us to observe a donor-dependent effect in addition to the previously described molecule-dependent effect (**Figure 5**). In the hydroxycarbamide-treated group, at baseline (S), in the controls, Firmicutes were dominant (50.3% of rarefied counts in donor A and 52.1% rarefied counts in donor B), with Bacteroidetes (27.9% rarefied counts in donor A and 36.7% rarefied counts in donor B) and Proteobacteria being less abundant (19.5% rarefied counts in donor A and 9.8% rarefied counts in donor B). In hydroxycarbamide, the variation in the 3 phyla during the experiment was comparable in the treated and control groups, as well as between the 2 donors (**Figure 5**).

In the daunorubicin group, at baseline (S), in the controls, Firmicutes were dominant (55.7% of rarefied counts in donor A and 48.4% rarefied counts in donor B) with Bacteroidetes (25.0% rarefied counts in donor A and 31.5% rarefied counts in donor B) and Proteobacteria being less abundant (16.4% rarefied counts in donor A and 16.9% rarefied counts in donor B).

Proteobacteria evolved comparably between the experiments and followed a constant increase through time in both donors and both molecules.

In the daunorubicin group, compared with baseline (S), we observed a decrease in the Bacteroidetes phylum from T1 in donor A. In donor B, Bacteroidetes evolved similarly throughout the experiment. A decrease in Firmicutes was observed in donor B compared with the variations in the control group (**Figure 5**).

**Figure 6:**
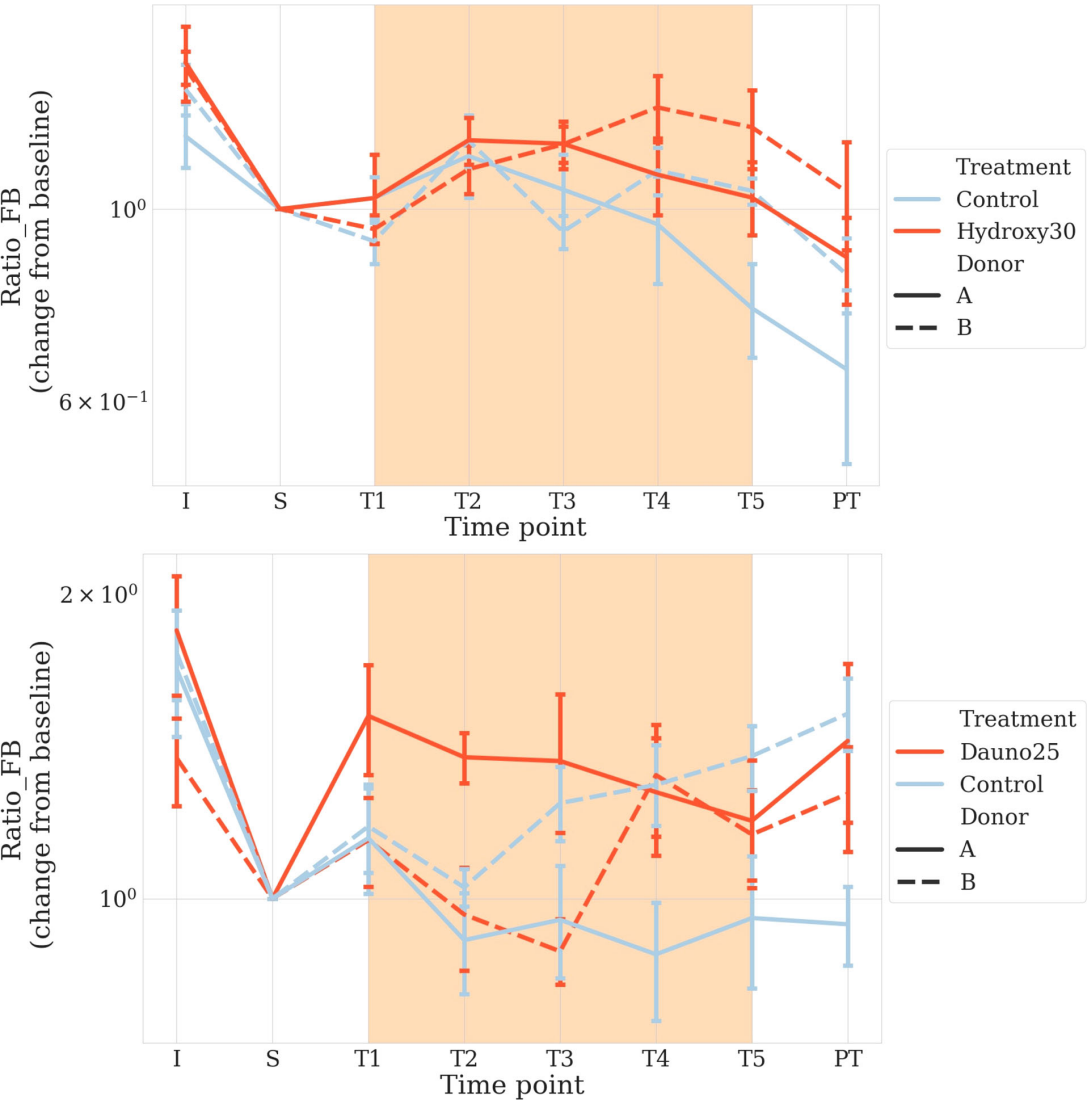
Firmicutes to Bacteroidetes ratio to early detect dysbiosis after treatment. Solid lines represent donor A, and dotted lines represent donor B Light blue represents the controls, and red represents the treated groups. Orange square represents the treatment period (average and 68% CI). The top figure represents hydroxycarbamide, and the bottom figure represents daunorubicin.

We also calculated the change in the Firmicutes/Bacteroidetes (F/B) ratio, which is admitted as an early dysbiosis biomarker (Ley et al., 2006; Montassier et al., 2014; Magne et al., 2020). When we used the variation of that ratio, we did not find any overall effects (using both PERMANOVA and a linear model with permutation *p* > 0.1) for both treatments. However, in both treatments, a donor-dependent effect was suggested with a signal in one of the two donors (hydroxycarbamide: donor B: aovp *p* = 0.01, lmp *p* = 0.04 and danurobicin, donor A aovp *p* = 0.004, lmp *p* = 0.004) (**Figure 6**). Because of this mixed signal, we resorted data to test the evolution in that ratio based on the change from baseline in each chamber. We used a similar approach to that used for the alpha and beta diversity analyses. We found that daunorubicin was associated with a marginally significant effect for donor B chambers (*p* = 0.03), but significant for donor A (*p* = 1e−4), and marginally significant for both *p* = 0.05. This is explained by the fact that the effect was inversed for each donor. Indeed, both had very different initial ratios, and through time, they converged towards an intermediate value. It seems daunorubicin slows down this convergence in both cases. Hydroxycarbamide showed a moderate impact for both donors (A and B: aovp: *p* = 0.0015, lmp: *p* = 0.001, A: aovp: *p* = 0.03, lmp: *p* = 0.04, B: aovp: *p* = 0.01, lmp: *p* = 0.01). A reduction in the ratio was observed in both donors.

## 4 Discussion

Relying on recent advances in oncology and the tight relation between the gut microbiota and human pathologies (Alexander et al., 2017; Aarnoutse et al., 2019; Lee and Jaeho, 2021), we aimed to use the MBRA model to evaluate the precise impact of 2 anticancer drugs on human fecal microbiota, from healthy donors, without any other therapeutics.

Hydroxycarbamide was the first cytotoxic anticancer drug prescribed orally, with an over 50,000 prescriptions in 2014 in France (https://www.iledefrance.ars.sante.fr/media/6333/download). It is a nonalkylating antineoplastic reversible DNA replication inhibitor (especially the S-phase), widely used in the treatment of hematologic pathologies such as chronic myeloproliferative disorders and in sickle cell disease (Singh and Xu, 2016). Its toxicity in bacteria has been shown *in vitro* on *Escherichia coli* (Davies et al., 2009), *Chlamydiae trachomatis* (Rosenkranz et al., 1973), and *Bacillus subtilis* (Wozniak and Simmons, 2021). This toxicity was mainly explained by high levels of oxidative stress induced by the drug (Singh and Xu, 2016). The mutagenic activity and the genotoxicity of this drug was confirmed in a *Salmonella*/microsome assay (Santos et al., 2011).

Daunorubicin is an anthracycline aminoglycoside used in the treatment of nonlymphocytic leukemia and acute lymphocytic leukemia in adults and children (Gong et al., 2015). It acts at various levels on cell replication, first through DNA damages (base pairs intercalating) and second by inhibiting the polymerase’s activity, therefore disturbing the regulation of gene expression. Its mutagenic/cytotoxic activity was evaluated in bacterial tests, hence its nonsurprising impact on bacteria (Babudri et al., 1984). Although it has an intravenous route of administration, its main elimination route is hepatobiliary (40%), suggesting a potential impact on the fecal microorganisms. Based on the current poor literature concerning these two drugs, there is no evidence confirming their implication in gut microbiota alteration and intestinal dysbiosis.

Using *in vitro* gut models to experiment with anticancer treatments is of great interest, but the equipment is often costly, and experiments are long. The MBRA system is performant, less costly compared to other existing *in vitro* models (Tang, 2019). One major advantage of this *in vitro* gut system is that no systemic immune response can interfere and modify our observations. Excluding this parameter is of major importance, especially to evaluate whether in absence of immune response, the drug’s impact remains identical.

Here, we show the impact of an anticancer drug, evaluated on gut microbiota, independently from its host, varies in a donor-dependent and molecule-dependent manner. With this system, minor effects can still be objectified. Indeed, we could show hydroxycarbamide, at 30 mg/L, did not show any major impact on the gut microbiota, and minor effects of daunorubicine at 25 mg/L could however be observed. Compared with the *in vivo* results on anticancer drugs-associated dysbiosis, we observe fewer dysbiotic signals (Montassier et al., 2015; Rashidi et al., 2019). Our experiments suggest that the impact observed *in vivo* is the result of several factors and not only the anticancer molecule. This places the host as a possible crucial actor in the observed gut microbiota disturbances *in vivo*, in the context of anticancer drugs.

Thus, before concluding on the impact of a drug on the gut microbiota and all of its subsequent consequences, several questions must be answered. When an effect is observed *in vivo*, no information is given on the major chain link responsible for this effect: disturbance could occur at the bacterial level, at the gut microbiota level, or at the host level. The strength of our study relies on the capacity of the MBRA system to analyze specifically the direct impact of a drug on the gut microbiota composition. Many studies have shown the impact of chemotherapy on intestinal dysbiosis, tumoral response, or toxicity. However, these conclusions seem hasty, as no data exist concerning the intermediate actors in this drug–microbiota link. Furthermore, many of the included patients in these studies received anticancer drugs and many other drugs concomitantly, including antibiotics, known as dysbiosis-inducing factors (Burdet et al., 2019).

Here, we studied specifically the relation between drug and gut microbiota composition, excluding all the confounding factors from the host (Rooks and Garrett, 2016), concomitant antibiotic treatments (Burdet et al., 2019), or the environment (Jin et al., 2017; Naimi et al., 2021). We demonstrated the effect of anticancer drugs on the gut microbiota depends on the donor hence the host, and on the molecule.

Among the many perspectives in oncology, the MBRA system could be an interesting tool to evaluate the impact of more drugs and combinations of drugs on the gut microbiota. Here, relying on previous works (Flores et al., 2014; Montassier et al., 2014; Brim et al., 2021), we measured the Firmicutes/Bacteroidetes ratio biomarker, allowing us to confirm the dysbiosis evidenced by the diversity metrics. This biomarker is easy to measure (Guo et al., 2008; Magne et al., 2020) and enables rapid screening for dysbiosis in a high-throughput manner. Ultimately, with a personalized medicine objective, analyzing the changes in the gut microbiota composition could help target patients at risk of poor therapeutic response to adapt the anticancer treatment specifically (Montassier et al., 2016; Alexander et al., 2017; Chaput et al., 2017; Malard et al., 2021).

In this work, we add evidence concerning the ability of the MBRA system to bring light into interindividual variations regarding anticancer drugs. To increase data on this topic, follow-up studies are needed on a larger cohort. Moreover, an effort will have to be made to better understand the relationship between the results obtained *in vitro* and the clinical response, which will make it possible to arrive at a personalized medicine allowing us to facilitate/improve the treatment of patients.

In conclusion, daunorubicin was shown to alter the diversity of the human gut microbiota at a higher level compared with hydroxycarbamide and in a donor-dependent manner. Whoever the donor, hydroxycarbamide did not show any significant impact on gut microbiota composition. Until now, no equivalent studies have been conducted, enabling this focus only on the gut microbiota and the anticancer drug. Our work enabled us to objectify the donor-dependent effect of a molecule and a molecule-dependent effect, excluding the main confounding bias inherent to the host and its environment. These disparities between donors should be further explored to understand the underlying mechanisms to this drug-associated dysbiosis. These host-dependent variations have yet to be shown in antibiotic resistance dynamics, in antibiotic–microbiota studies, and now in anticancer-drug–microbiota. All these elements confirm the importance of personalized medicine in the future, for which the MBRA model seems particularly valuable.

## Data Availability Statement

The data presented in the study are deposited in the NCBI SRA repository, accession numbers PRJNA841222 and PRJNA841052.

## Ethics Statement

In agreement with the INSERM ethics regulation, and with the declaration of Helsinki, all volunteer and anonymous donors received clear information and consented.

## Author Contributions

CAH, MM, BG, and AB performed the laboratory work. CAH, LV, AB, and OT conceived the study, analyzed the data, performed statistical analyses, and wrote the manuscript. AB and OT supervised the study. BC, and SB helped in the conception of the study and revised the manuscript. TS and PC advised the choice of the drugs, of their concentration, and provided them ready to use in sterile packagings. All authors listed have made a substantial, direct, and intellectual contribution to the work and approved it for publication.

## Funding

CAH is currently supported by funds from the ARC Foundation (Grant No. DOC20190509066) for a PhD. The Q.E.M Team currently works with FRM funding (EQU201903007848). We also relied on the ANR GeWiEp (ANR-18-CE35-0005-0) funding for the equipment. BC’s laboratory is supported by a Starting Grant from the European Research Council (ERC) under the European Union’s Horizon 2020 research and innovation program (grant agreement No. ERC-2018-StG-804135), a Chaire d’Excellence from IdEx Université de Paris - ANR-18-IDEX-0001, an Innovator Award from the Kenneth Rainin Foundation, an ANR grant EMULBIONT ANR-21-CE15-0042-01, and the national program “Microbiote” from INSERM.

## Conflict of Interest

The authors declare that the research was conducted in the absence of any commercial or financial relationships that could be construed as a potential conflict of interest.

## Publisher’s Note

All claims expressed in this article are solely those of the authors and do not necessarily represent those of their affiliated organizations, or those of the publisher, the editors and the reviewers. Any product that may be evaluated in this article, or claim that may be made by its manufacturer, is not guaranteed or endorsed by the publisher.

## Abbreviations

MBRA, MiniBioReactor Array; PCoA, principal coordinate analysis
